# Meta‐analysis of goal‐directed fluid therapy using transoesophageal Doppler monitoring in patients undergoing elective colorectal surgery

**DOI:** 10.1002/bjs5.50229

**Published:** 2019-10-22

**Authors:** A. Feldheiser, R. Casans Francés, M. Stopfkuchen‐Evans

**Affiliations:** ^1^ Department of Anesthesiology and Operative Intensive Care Medicine (CCM, CVK), Charité ‐ Universitätsmedizin Berlin Berlin Germany; ^2^ Department of Anaesthesia and Pain Therapy, Hospital Universitario Infanta Elena Valdemoro Madrid Spain; ^3^ Department of Anesthesiology, Perioperative and Pain Management Brigham and Women's Hospital, Harvard Medical School Boston Massachusetts USA

We read with great interest the meta‐analysis published by Rollins and colleagues^1^. The study found no benefit for oesophageal Doppler monitoring (ODM) *versus* conventional fluid management in patients undergoing abdominal surgery, and was methodologically well performed.

However, the study selection raises concerns that impact the result substantially. The included study by Brandstrup *et al*.^2^ compares a perioperative zero‐balance group with intraoperative ODM. The study found no differences between the groups. However, zero‐balance therapy has to be considered as intervention‐based, given specialized beds being able to determine patient weight without mobilization, which was associated with a higher perioperative workload *versus* the ODM. The study was considered a success for ODM, and therefore should not be included in a meta‐analysis comparing ODM with conventional care.

Furthermore, the most influential study regarding ODM by Calvo‐Vecino and co‐workers^3^, with 420 patients, was not included in the analysis. This would have increased the sample size by 37·7 per cent. The study was performed as a multicentre RCT within an enhanced recovery after surgery pathway in patients undergoing abdominal surgery, and showed a reduction in postoperative morbidity, especially surgical‐site infection (SSI).

Consequently, the meta‐analysis should be modified by exclusion of the study by Brandstrup and colleagues^2^ and inclusion of that by Calvo‐Vecino *et al*.^3^. As the Brandstrup study has significant weight and the Calvo‐Vecino study is the largest to date, a reduction in morbidity and SSI is the most likely effect of correcting the meta‐analysis. With that modification, the meta‐analysis would be consistent with the actual literature and previously published meta‐analyses on the topic^4^, ^5^.

## Disclosure

The authors declare no conflict of interest.
